# Adherence to medication administration guidelines among nurses in a health facility in South-West Nigeria

**DOI:** 10.11604/pamj.2021.40.56.27562

**Published:** 2021-09-22

**Authors:** Cecilia Bukola Bello

**Affiliations:** 1 Department of Nursing Science, College of Medicine and Health Sciences, Afe Babalola University Ado-Ekiti, Ekiti State, Nigeria

**Keywords:** Medication administration, medication guidelines, adherence, patient safety, nurses

## Abstract

**Introduction:**

medication error has become a global problem. Medication administration that is error free is important in achieving positive outcomes in patient’s care. This study assessed adherence to medication administration guidelines among nurses in a health facility in South-West Nigeria.

**Methods:**

a cross-sectional descriptive study was carried out on 75 nurses involved in oral medication administration. Data was collected using direct observation method with an observational checklist developed from literature. Data analysis was done using frequency, percentage, Mean and Standard Deviation. Test of relationship was carried out using Kruskal-Wallis Test and Mann Whitney Test at 0.05 (p<0.05) level of significance.

**Results:**

almost an average (49.3%) of participants did not provide information about the medication. More than 1/3^rd^ (38.7%) did not perform right assessment where necessary. The majority (76.0%) did not serve correct medication. Overall level of non-adherence was 48%. Adherence to medication administration guidelines was significantly related to age (χ^2^ = 9.673, p<0.05), marital status (χ^2^ = 9.426, p<0.05), years of experience (U=404.000 Z=2.7622, p<0.05), type of shift (χ^2^ = 6.314, p<0.05), nurses-patient ratio (χ^2^ = 11.598, p<0.05).

**Conclusion:**

some nurses did not adhere strictly to the guidelines of medication administration. Adherence to medication administration guidelines was related to age, marital status, years of experience, type of shift and nurse-patient ratio. Poor adherence to medication administration guidelines may jeopardize patient safety. There is need for development of a universal medication procedure/protocol and continuous education of nurses on medication administration practices.

## Introduction

Medication administration is one of the most important duties of a professional nurse, medications are usually served on the order of physicians and dentists [1]. Oral medication administration method is more common generally because it is convenient and cost-effective [2]. Medication administration procedure is a complex process that is carried out step by step [1] many times in a day [3]; which increases the opportunity for error. Ensuring safety is an important professional responsibility of nurses [4, 5]. Patient safety is a strategic goal and core value in nursing practice, while medication administration that is error free is an important process of achieving positive outcomes in patient´s care. Over the years, there are a set of guidelines that nurses must follow to ensure medication administration safety, which is referred to as the “rights”of medication administration [6]. Observation of the “rights”of medication administration are important aspect of the medication administration process, which is recognized as the basic standard for safe medication practice [1-3].

Patient´s safety can be achieved through good practices during medication administration. However, there are reports of poor adherence of health professionals to safety guidelines during medication administration [7, 8]. This reduces the quality of care and put patients at risk. Medication administration error is one of the most popular causes of morbidity and mortality in the clinical settings [9]. Studies have identified common factors responsible for medication administration errors. With the growing awareness of the role that medication errors play in preventable deaths, more attention should be given to nurse´s adherence to medication administration guidelines as this play an important role in ensuring medication safety. Medication administration is the last step in the medication use process and the nurse administering that medication provides the final line of defence for the patient [1, 10]. However, despite these assertions, very little is known about the extent to which nurses adhere to safety guidelines to prevent medication error in many of the health care settings in Nigeria. In view of the current shortage of nurses in health facilities in Nigeria and its negative effect on nurse´s job performance, this study examines the extent of adherence to medication administration guidelines during oral medication administration procedure and identifies related factors in a secondary health facility in Ondo State, South-West, Nigeria.

## Methods

**Research design:** this study adopted a descriptive cross-sectional design. A direct observational method was used, nurses were observed during oral medication administration.

**Study setting:** this study was conducted in State Specialist Hospital Akure, Ondo State, which is located in the South-West of Nigeria. It´s a secondary health facility with ten wards. Each ward has a maximum of 30 beds and minimum of 15 beds.

**Population and selection of participants:** the target population were professional nurses in State Specialist Hospital Akure. A purposive sampling technique was used to select six out of ten wards where patients were admitted and nurses were involved in direct patient´s care. The wards were considered based on factors such as type of cases (surgical/medical/obstetrical or gynaecological wards), patients sex (male/female wards), and patients age (children/adults wards). This was to determine the patterns of practice in these wards. Accidental sampling technique was used to select nurses. Nurses that were on duty and assigned for medication administration in all shifts were included in this study. A good sample size from a known population is usually 10%, but due to the few number of nurses in each ward;more than 50% of nurses in selected wards were involved. This is to allow a good representation of nurses in selected wards. A total of seventy-five (75) observations were carried out.

**Instrument for collecting data:** a 14-item observational checklist was developed from the recommended step-by-step procedure of medication administration [1]. The tool was arranged into 2 sections. The first section contained the socio-demographic characteristics of participants, this included: ward name, age, gender, marital status, educational background, years of experience, name of shift, and number of nurse´s ratio to number of patients on admission. The second part contained the observation checklist. This was classified into 4 categories (medication reconciliation/verification, patient identification/communication, medication administration and documentation). The checklist was given to 3 clinical nurse experts to ensure content validity and also pilot tested using 5 nurses in wards not included in the study. Items carried out by the nurse during the medication administration procedure were marked as adhered to and scored 1 while items not carried out were marked not adhered to and scored 0.

**Method of data collection:** the nurses assigned for administration of medication were observed during the procedure in six wards within 6 months (August 2018 till January 2019). Observation was limited to oral medication administration only. A direct observation was carried out. Participants were informed that they were being observed, but further details were not shared. In order to reduce the Hawthorne effect that is peculiar to direct observation, participants were observed in the ward setting, which is considered natural. Nurses were already used to the researcher who came to the ward many times to give assistance in various procedures two weeks before data collection. The checklist was ticked as appropriate as the procedure was ongoing from one patient to the other till the end of the procedure. The socio-demographic characteristics of the nurse assigned for medication was collected after the procedure.

**Ethical considerations:** ethical approval for this study was obtained from Hospital´s management Board Ondo State with a reference number G23/29. Then, permission to conduct the study was collected from the Head of nursing services of the facility and ward heads. Ward heads were informed about the observation and the purpose. Individuals to be observed were informed that they were being observed, but details about the observation were not revealed. Informed consents were obtained. Data collected were kept securely to ensure confidentiality.

**Data analysis:** data was analysed using the Statistical Package for Social Sciences, version 20. Frequency, percentages, mean and standard deviation were used to calculate participants´ adherence to medication guidelines. Descriptive data were also presented in charts. Significant association was determined using Kruskal-Wallis and Mann Whitney Chi-square.

## Results

Table 1 showed the socio-demographic characteristics of participants. Equal number of participants (16%) were involved from each ward except postnatal ward with a higher number (20%). Mean age and standard deviation of participants was 39.2±8.1 years, while majority (96%) of participants were females. More than half (62.7%) of participants had a Bachelor of nursing science degree. The mean and standard deviation of participant´s years of experience was 13.6±10.1 years. Nurse patient ratio was majorly 1 nurse to 7 patients (36.0%).

**Table 1:** socio-demographic characteristics of participants

Socio-demographic variables		Frequency (n= 75)	Percentage (100%)
**Name of ward:**	Male medical ward	12	16.0
Ward	Female medical	12	16.0
	Male surgical ward	12	16.0
Ward	Female surgical	12	16.0
	Children’s ward	12	16.0
	Postnatal ward	15	20.0
**Age:[39.2±8.1 years]**	<31 years	6	8.0
	31-40 years	45	60.0
	41-50 years	15	20.0
	51+ years	9	12.0
**Sex**	Male	3	4.0
	Female	72	96.0
**Marital status**	Single	3	4.0
	Married	69	92.0
	Widowed	3	4.0
**Educational background**	Diploma certificate	28	37.3
	Bachelor of nursing science	47	62.7
**Years of experience: [13.6±10.1 years] <11 years**		48	64.0
years	11-20	27	36.0
**Type of shift**	Morning	42	56.0
	Afternoon	18	24.0
	Night	15	20.0
**Nurse/patient ratio**	1 nurse to 2 patients	3	4.0
	1 nurse to 3 patients	15	20.0
	1 nurse to 4 patients	15	20.0
	1 nurse to 5 patients	9	12.0
	1 nurse to 6 patients	6	8.0
	1 nurse to 7 patients	27	36.0
		**75**	**100.0**

Table 2 showed adherence of participants to medication administration guidelines. The majority of participants adhered to medication administration guidelines, however, about an average (49.3%) of participants did not provide information about the medication. Also, more than 1/3^rd^ (38.7%) did not perform right assessment where necessary. The majority (76.0%) did not serve correct medication.

**Table 2:** adherence of participants to medication administration guidelines

Medication administration guidelines	Adherent n (%)	Non-adherent n (%)	Mean	SD
1 Check for new orders	63 (84.0)	12 (16.0)	0.84	0.319
2 Check orders are correctly transcribed	70 (93.3)	5 (6.7)	0.93	0.251
3 Correct client identification	72 (96.0)	3 (4.0)	0.96	0.197
4 Receive feedback on the medication	38 (50.7)	37 (49.3)	0.51	0.502
5 Provide information about medication	54 (72.0)	21 (28.0)	0.72	0.452
6 Perform right assessment	46 (61.3)	29 (38.7)	0.61	0.490
7 Perform 3 checks before serving medication	66 (88.0)	9 (12.0	0.88	0.327
8 Use accurate measuring container	66 (88.0)	9 (12.0)	0.88	0.327
9 Correct dose of medication given	75 (100.0)	0 (0.0)	1.0	0.000
10 Correct medication served	18 (24.0)	57 (76.0)	0.24	0.430
11 Medication served at the right time	72 (96.0)	3 (4.0)	0.96	0.197
12 Observed patient after serving medication	72 (96.0)	3 (4.0)	0.96	0.197
13 Immediate documentation	75 (100.0)	0 (0.0)	1.0	0.000
14 Put her signature in her documentation	69 (92.0)	6 (8.0)	0.92	0.273

**Figure 1:**
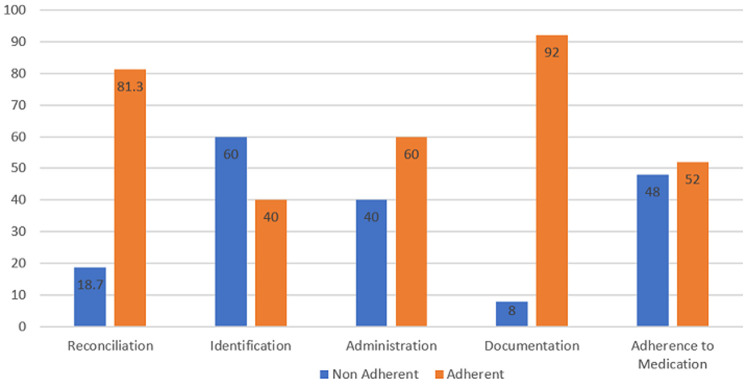
prevalence of adherence/non-adherence to medication administration guidelines

Figure 1 showed the prevalence of adherence/non-adherence to medication administration guidelines based on classification into reconciliation, identification, administration and communication and documentation. Identification/communication had the highest level of non-adherence, with 60%. It also showed overall result of adherence/non-adherence. A total of 48% of participants did not adhere to medication administration guidelines.

Table 3 showed adherence to medication administration guidelines and relationship with the socio-demographic characteristics of participants. Result showed that participants who were 41 years and above had higher mean score (12.0). Male participants also had higher mean score (12.3) compared to female participants. The level of adherence was very high among participants who were widows (13.0). There is a slight difference in mean score based on years of experience, participants with more than 11 years of work experience had slightly higher mean score than participants with less than 11 years of work experience. Participants on morning shift had higher mean score (11.8). The level of adherence to medication administration varies with the number of nurses to patient´s ratio; mean score of participants reduces with the ratio of 1 nurse to higher number of patients. The ratio of 1 nurse to 2 patients had the highest Mean score (13.0). Further analysis showed that adherence to medication administration guidelines is significantly related to age (χ^2^= 9.673, df =5 p<0.05), marital status (χ^2^= 9.426, df =2, p<0.05), years of experience (U=404.000 Z=2.7622, p<0.05), type of shift (χ^2^= 6.314, df =2 p<0.05), number of nurses to patient´s ratio (χ^2^= 11.598, df =5 p<0.05).

**Table 3:** adherence to medication administration guidelines and relationship with the socio-demographic characteristics of participants

n-75	Adherence to medication administration guidelines							
Socio-demographic variables	Mean score	SD	Mean rank	Min	Max	Kruskal-Wallis/Whitney Chi-square	Df/z score	P value (<0.05)
**Name of ward**						6.141	5	0.293
Male medical	11.5	0.7	37.63	10.0	13.0			
Female medical	10.8	1.8	29.96	8.0	14.0			
Male surgical	11.9	2.0	48.21	7.0	14.0			
Female surgical	11.2	1.7	36.54	8.0	13.0			
Children’s	11.0	1.4	32.08	8.0	13.0			
Postnatal	11.7	1.4	42.47	8.0	13.0			
**Age:** <31 years	11.3	1.2	34.33	10.0	10.0	9.673	3	0.022
31-40 years	11.1	1.5	32.72	7.0	7.0			
41-50 years	12.0	2.0	51.20	8.0	8.0			
>50 years	12.0	0.8	44.83	11.0	11.0			
**Sex:** male	12.3	0.5	50.67	12.0	13.0	70.000	1.054	0.2972
Female	11.3	1.6	37.47	7.0	14.0			
**Marital status**						5.331	1	0.021
Single	9.3	0.5	9.83	9.0	10.0			
Married	11.4	1.5	38.14	7.0	14.0			
Widowed	13.0	0.0	63.00	13.0	13.0			
**Education**						606.500	0.579	0.563
Diploma	11.2	1.7	36.16	7.0	14.0			
BNSc	11.4	1.5	39.10	8.0	13.0			
**Years of Experience**								
<11 years	11.1	1.5	32.92	7.0	14.0	404.000	2.762	0.006
>11 years	11.9	1.6	47.04	8.0	13.0			
**Shift:** morning	11.8	1.4	43.46	8.0	14.0	6.314	2	0.043
Afternoon	10.9	1.6	31.22	7.0	13.0			
Night	10.8	1.6	30.83	8.0	13.0			
**Nurse/patient ratio**						11.598	5	0.041
1 to 2 patients	13.0	0.0	63.0	13.0	13.0			
1 to 3 patients	10.4	1.8	26.43	8.0	13.0			
1 to 4 patients	12.0	0.7	44.77	11.0	13.0			
1 to 5 patients	11.7	0.9	40.94	11.0	13.0			
1 to 6 patients	11.0	0.8	28.33	10.0	12.0			
1 to 7 patients	11.4	1.8	39.06	7.0	14.0			

## Discussion

The main aim of this study was to assess nurse´s adherence to medication administration guidelines. A total of 75 nurses were observed in six wards. The majority of participants were females within 31-40 years of age with less than 11 years of work experience. This is similar to participants used in previous studies [11, 12]. Findings from this study showed that more than 1/3^rd^ of participants did not provide any information to the patient before medication administration. Such information includes the name of the drug, its purpose, and side effects that the patient may experience. This is similar to findings in previous studies [13, 14] that reported that only 13.4% of nurses explained the purpose of medication to patients and 35.9% of nurses did not tell patients names of the medication respectively. One of the essential guidelines to follow to ensure safety during medication administration is to educate patients and provide important information about their medication. This helps patient to know what to expect and quickly report issues such as adverse effects. More than 20% of nurses did not listen to feedback from patients.

Most studies on adherence to medication administration guidelines did not include this in their findings. It is essential to listen to patient for feedback on the medication they receive, such feedback may include complaints about side effect, adverse effect or discomfort about medication. This is important as such feedback may necessitate considering alternatives or adjusting doses. Another finding from this study showed that more than 1/3^rd^ of participants did not perform right client assessment before medication administration, such assessments include previous history of allergy, checking of pulse, blood pressure or presence of pain, bowel movement, vomiting etc. This is similar to findings in a previous study [13] where only 5.7% of nurses asked about the history of allergy to a medicine before administration.

Furthermore, this study reported that 76% of nurses did not serve correct medicine (one or two medicines were erroneously omitted or medicine that were not included, such as meant for another time were administered). This is similar to findings in a study in Addis Ababa [14] that reported that 16.4% of nurses did not administer the right medicine. Serving the wrong medication may alter the expected effect of the medicine and in other cases may be responsible for resistance and toxicities [14]. This study reported that all nurses documented medication served while only few did not put their signature. This is consistent with a study that reported correct documentation of medications by majority [15]. But contrary to these findings, a study reported that 52% of nurses did not document necessary information [14]. Lack of documentation may cause duplication of medication to patient, prevents continuity of care and increases the risk of errors. A prevalence of 48% for non-adherence to medication administration guideline was revealed by this study. This is considered a high prevalence and serves as potential risk for patient´s safety and needs prompt intervention. A previous study [11] reported 34.5% prevalence of non-adherence. Participants with higher age adhered to medication administration guidelines and this is significant. A participant´s age can be said to be related to their years of work experience; younger age will have lesser years of work experience. A higher adherence to medication administration guidelines among nurses with higher age can be explained in relation to higher years of work experience. This is similar to findings in a study. In corroboration to this finding, a study in Ethiopia [16] reported an association of medication error with age of nurses.

Furthermore, this study revealed relationships between adherence to medication administration guidelines and type of shift [17] reported a significant relationship of adherence to medication administration guidelines with type of shift. Lack of sleep during the night shift may result in poor concentration and inability to adhere to medication administration guidelines. In addition, this study reported a relationship between adherence to medication administration guidelines and nurse to patient ratio. A ratio of 1 nurse to higher number of patients translate to increase in workload. In a literature review [18, 19], reported that being overworked was the most prevalent factor responsible for medication administration error. As patient increases, nurse´s workload increases; this may lead to stress and fatigue and may reduce the willingness to adhere to safety guidelines during medication administration.

**Limitation of the study:** this study was conducted in just one health facility and only in few wards in the facility; this limits the ability to generalize findings of this study to other wards and other health institutions in South-West, Nigeria.

## Conclusion

This study concludes that some nurses do not adhere strictly to the guidelines of medication administration. Adherence to medication administration guidelines was significantly associated with age, marital status, years of work experience, type of shift and nurse-patient ratio. Poor adherence to medication administration guidelines expose patient to risk of medication error and may jeopardize patient safety and results in poor health outcomes. Adequate number of nurses on duty in relation to number of patients is essential for adherence to safety guidelines. There is need for development of a universal medication procedure/protocol and continuous education of nurses on medication administration practices.

### What is known about this topic

- Non-adherence to medication administration guidelines results in medication errors;
- Medication error is a global problem responsible for disabilities and deaths;
- Poor adherence to medication administration guidelines from previous studies and settings outside Nigeria.

### What this study adds

- Findings revealed that some nurses do not adhere strictly to the guidelines of medication administration while performing the procedure.
- Findings revealed that nurses´ age, years of experience, shift and nurse/patient ratio were significantly associated with non-adherence to medication administration guidelines.
